# Disruption of the blood–brain barrier after generalized tonic-clonic seizures correlates with cerebrospinal fluid MMP-9 levels

**DOI:** 10.1186/1742-2094-10-80

**Published:** 2013-07-05

**Authors:** Ya-Jun Li, Zheng-Hai Wang, Bei Zhang, Xiao Zhe, Ming-Jue Wang, Shao-Ting Shi, Jing Bai, Tao Lin, Chang-Jiang Guo, Shi-Jun Zhang, Xiang-Li Kong, Xing Zuo, Hang Zhao

**Affiliations:** 1 Department of Neurology, The Affiliated Hospital of Xi’an Medical University, No. 48, West Fenghao Road, Xi’an 710077, Shaanxi Province, China; 2 Department of Neurology, The General Hospital of Ningxia Medical University, Ningxia, China

**Keywords:** Cerebrospinal fluid, Seizure, BBB, Metalloproteinase-9, Leukocytes

## Abstract

**Background:**

Increasing evidence suggests seizures cause blood–brain barrier (BBB) dysfunction including decreased seizure threshold and higher onset potential of future seizures. However, the mechanisms underlying BBB damage in seizures remains poorly understood. Evidence in human and animal models shows BBB disruption is associated with activation of matrix metalloproteinase-9 (MMP-9) after cerebral ischemia and inflammation. The objective of this study was to determine whether MMP-9 concentrations in cerebral spinal fluid (CSF) are associated with BBB disruption in patients after epileptic seizures.

**Methods:**

Thirty-one patients with generalized tonic-clonic (GTC) seizures were included in the study: 20 had recurrent GTC seizures (RS), and 11 had a single GTC seizure (SS) episode. Twenty-five adult non-seizure patients were used as controls. CSF samples were collected by lumbar puncture within 24 h after seizure cessation (range: 3–15 h, mean 6.2 h). CSF MMP-9 levels were determined by an enzyme-linked immunosorbent assay (ELISA). MMP enzyme activity was measured by gelatin zymography. The CSF/serum albumin ratio (albumin quotient, QAlb) was used as a measure of blood–brain barrier permeability.

**Results:**

We found significantly higher CSF MMP-9 concentrations in seizure patients compared with controls (*P* < 0.001). CSF MMP-9 levels and QAlb values were higher in RS patients compared with SS and controls. Moreover, CSF MMP-9 concentration showed strong correlation between QAlb values (r = 0.76, *P* < 0.0001) and between CSF leukocyte counts (r = 0.77, *P* < 0.0001) in patients after seizures. Gelatin zymography showed MMP-9 proteolytic activity only in GTC seizure patients.

**Conclusions:**

Our results suggest MMP-9 plays a role in BBB dysfunction, characterized by invasion of leukocytes into the CSF during seizures.

## Background

The blood–brain barrier (BBB) is the most important vascular barrier of the central nervous system (CNS). Due to its unique structure, the BBB limits penetration of a variety of harmful substances from the blood into the brain, while also supplying the brain with nutrients required for proper function. In recent years, animal models and human clinical data have described a central role for vascular integrity, specifically the permeability of the BBB, as an important mediator of brain damage, including the delayed appearance of neuronal dysfunction and death [1-4]. Studies show BBB dysfunction is common following traumatic, ischemic or infectious brain insults, and it may last from several days to weeks and even years after the acute event [5,6]. Recently, clinical and experimental data have correlated primary BBB lesions with seizures and epileptogenesis. Experimental studies have demonstrated a rapid increase in BBB permeability in animals experiencing long-lasting seizures, especially status epilepticus (SE). Studies in human epileptic patients are consistent with the animal data, showing an increase in BBB permeability during seizures [7,8]. Pentylenetetrazole-induced seizures cause BBB disruption, allowing permeation of blood-borne large molecules, such as albumin, into the CNS [9,10]. BBB disruption decreases the seizure threshold and facilitates the onset of seizures, and it is independent of the fact that such disruption is associated with or a result of the seizure itself [11]. The mechanisms underlying the BBB disruption in seizures and epileptogenesis are not entirely clear. There is evidence in human and animal stroke models that BBB disruption is associated with activation of matrix metalloproteinases (MMPs) [12-14].

MMPs are a family of zinc-dependent endopeptidases that are subdivided according to their substrate affinities for different components of the extracellular matrix. Among the various MMPs, metalloproteinase-9 (MMP-9), also known as gelatinase B, is thought to play an important role in BBB disruption after cerebral ischemia and inflammation [12,15-17]. MMP-9 degrades collagen IV, a major component of the basement membrane of the cerebral epithelium that is responsible for the integrity of BBB. The activity of MMP-9 is further controlled by the specific tissue inhibitor TIMP-1. However, the link between CSF MMP-9 levels and the presence of BBB disruption in patients after seizures has not yet been investigated in humans. Our study aimed to determine whether MMP-9 can be measured in CSF of patients after epileptic seizures and whether there is a correlation between CSF MMP-9 levels and QAlb values in patients after epileptic seizures.

## Methods

### Patient recruitment

The study was performed at the Department of Neurology of the Affiliated Hospital of Xi’an Medical University. Thirty-one patients with either tonic-clonic or partial secondarily generalized seizures were included in the study: 20 had recurrent GTC seizures (RS), and 11 had a single GTC seizure (SS) episode confirmed by an eyewitness that occurred within 3 h before admission. On admission to the emergency ward, all seizure patients received standard intravenous treatment with diazepam (dose range 10–20 mg) to stop seizure activity. Then patients were admitted to a neurologic ward for further evaluation. All patients underwent a standard diagnostic workup including neurologic examination and blood biochemical assessment. Ten patients presented the first-ever epileptic seizure and underwent further examinations including electroencephalography (EEG) and either a computed tomography (CT) or magnetic resonance imaging (MRI) scan, and the final diagnosis of epilepsy was confirmed (8 with cryptogenic localization-related epilepsy and 2 with idiopathic generalized epilepsy). Twenty-one patients already had an epilepsy diagnosis: 6 with cryptogenic localization-related epilepsy and 15 with symptomatic localization-related epilepsy with secondary generalization. Those patients presenting apparent symptomatic etiology of seizures, that is, electrolyte disturbances, defined metabolic causes, drug intoxication, infections, trauma or abnormal CT or MRI suggestive of acute brain diseases were excluded. All patients were studied within 24 h after the seizure. Epilepsy was diagnosed and classified according to the criteria proposed by the International League Against Epilepsy in 2011 [18]. Seizures and epileptic syndromes were classified according to the ILAE diagnostic criteria.

The control samples were obtained from 25 adult patients (mean age 39 ±13.6 years; range 16–56 years) by lumbar puncture and examined to exclude those with neurological disease, including nonspecific symptoms without diagnosed organic neurologic disease (*n* = 7), peripheral nervous system disorders (*n* = 4), acute headache (*n* = 5), spontaneous intracranial hypotension (*n* = 3), compressive radiculopathy (*n* = 4) and primary dementia (*n* = 2). All controls had normal neurological examination and normal CSF results on routine analysis.

All epileptic patients and controls were fully informed of the risks and potential benefits of the CSF examination as part of the diagnostic workup. Informed consent to participate in the study was obtained from each subject (or from the next of kin if the patient was incapable). Five patients were considered for the study but did not agree to undergo lumbar puncture and were not included in the study. The study protocol was approved by the Ethics Committee of the Affiliated Hospital of Xi’an Medical University, and all investigations were done in accordance with the criteria of the Declaration of Helsinki.

### CSF/serum sampling and biochemical analysis

CSF samples were taken between 10 a.m. and 5 p.m. by lumbar puncture from the L3/L4 or L4/L5 inter-vertebral space. Lumbar puncture was performed within 24 h after seizure cessation (range: 3–15 h, mean 6.2 h). CSF samples that were not clear or initially contained blood with gradual clearing were excluded from analysis. The first 2 ml of CSF was used for routine clinical tests and the subsequent 0.5 ml for our study. The CSF white blood cell (WBC) count, differential leukocyte count, total protein concentration, glucose (Glu) and chloride (CL) values were determined by standard methods immediately after lumbar puncture. Cytology of CSF cells was also performed. CSF and peripheral blood samples were collected simultaneously and centrifuged for 10 min at 2,500 g; 500 μl of the cell-free samples was immediately frozen and stored at −80°C until analysis. Concentrations of MMP-9 were measured by commercial enzyme-linked immunosorbent assay (ELISA) kits (R & D Systems, Minneapolis, MN) performed according to the manufacturer’s instructions. Optical density values were determined with a microplate reader set to 450 nm.

### Measurement of the QAlb value

Quantitative determination of albumin in the CSF and serum of all specimens were measured by a commercial kit (BioAssay Systems, Hayward, CA). The CSF/serum albumin ratio (albumin quotient, QAlb) was used as a measure of blood–brain barrier permeability. QAlb was calculated using the formula: QAlb = CSFAlb/serum Alb × 10^3^.

### Gelatin zymography

Activity of MMP-2 and MMP-9 enzymes in CSF samples was determined by gelatin zymography as previously described [16]. Briefly, activity of MMPs was analyzed by modified sodium dodecyl sulfate-polyacrylamide gel electrophoresis. Stacking gels contained 4% polyacrylamide, and separating gels contained 12.5% polyacrylamide and 0.1% gelatin. The 2 ml CSF was centrifuged at 10,000 × *g* for 15 min at 4°C to remove debris. Protein contents of supernatants were then mixed with an equal volume of 2× non-reducing sample buffer, and 25 μl was loaded per well. The gels underwent electrophoresis at 90 V and 4°C in running buffer (25 mM Tris, 250 mM glycine, 0.1% sodium dodecyl sulfate) until the bromophenol blue marker dye reached the bottom of the gel. After electrophoresis, the gel was transferred into a 2.5% Triton X-100 wash for 1 h at room temperature. After decanting the washing solution, the gel was equilibrated with developing buffer (50 mM Tris–HCl, pH 7.5, 200 mM NaCl, 5 mM CaCl2, 0.02% Brij-35, 0.01% NaN3) for 30 min at room temperature with gentle agitation. The gel was then placed in fresh developing buffer and incubated at 37°C for 18 h. The gel was stained for 1 h with 0.25% Coomassie Brilliant Blue R-250 (Sigma, St. Louis, MO) and was destained in 15% methanol/7.5% acetic acid. MMP activity was detected as white bands of lysis against the Coomassie blue-stained gel. The CSF zymography experiments were performed in triplicate. The gels were digitalized and the integrated density of the bands expressed as arbitrary units. The intensities of the gelatinolytic bands corresponding to MMP-9 and MMP-2 were calculated using the open-access software Image J 1.46r (National Institutes of Health, Bethesda, MD, USA; http://rsb.info.nih.gov/ij).

### Statistical analysis

Data are presented as mean ± SD for normally distributed and non-parametric data. Student's t-test and Mann–Whitney U-test were used for comparison of normally distributed and non-parametric data, respectively, between groups. ANOVA and Kruskal-Wallis test were used for comparison of normally distributed and non-parametric data, respectively, between multiple groups. Spearman's correlation coefficient was used to correlate variables in the groups studied. The calculations were performed with GraphPadInStat version 3.05 software (GraphPad Software, Inc., San Diego, CA, USA). For all tests, *P* < 0.05 was considered significant.

## Results

### Demographics of study participants

Demographic details of patients with epilepsy and controls are presented in Table 1. Thirty-one seizure patients and 25 controls provided data for the study. The mean age of the seizure patients was 42.5 (range, 20–68) years, and 16 (44%) were women. Among the controls, mean age was 38.7 (range, 22–71) years, and 12 (48%) were women. There were no differences between the two groups for these demographics. Twelve patients were receiving monotherapy, 9 polytherapy (the mean number of medications 2.3) and 10 patients no medication.

**Table 1:** Clinical characteristics of seizure patients and control subjects

**Variable**	**Patient**	**Control**	***P *value**
Number (*n*)	31	25	
Current age (years)	42.5 (15.1)	38.7 (17.6)	*P* > 0.05
Age at onset of epilepsy (years)	23.3 (14.8)		
Gender (M:F), *n*	20/16	13/12	*P* > 0.05
Red blood cells (10^12^/l)	4.3	4.5	*P* > 0.05
White blood cells (10^9^/l)	5.9	6.2	*P* > 0.05
**Focus of seizure**			
Temporal, *n* (%)	13 (41.9)		
Extratemporal, *n* (%)	18 (58.1)		
**Type of seizure**			
Single GTC seizure, *n* (%)	11 (35.5)		
Repetitive GTC seizure, *n* (%)	20 (64.5)		
Epilepsy duration (years)	6.3 (1–18)		
Frequency of seizures (month)	1.5 (1–5)		
Number of seizures	3.3 (1–10)		
Total duration of seizure (min)	8.3 (2–13)		
Time to sampling (h)	6.2 (3–15)		
Anti-epileptic drugs used	21 (67.7)		
No treatment	10 (32.3)		

Mann–Whitney *U*-test for continuous variables; cross tabulations and chi-square-test for categorical variables.

### MMP-9 concentrations and QAlb values are higher in seizure patients

**Figure 1:**
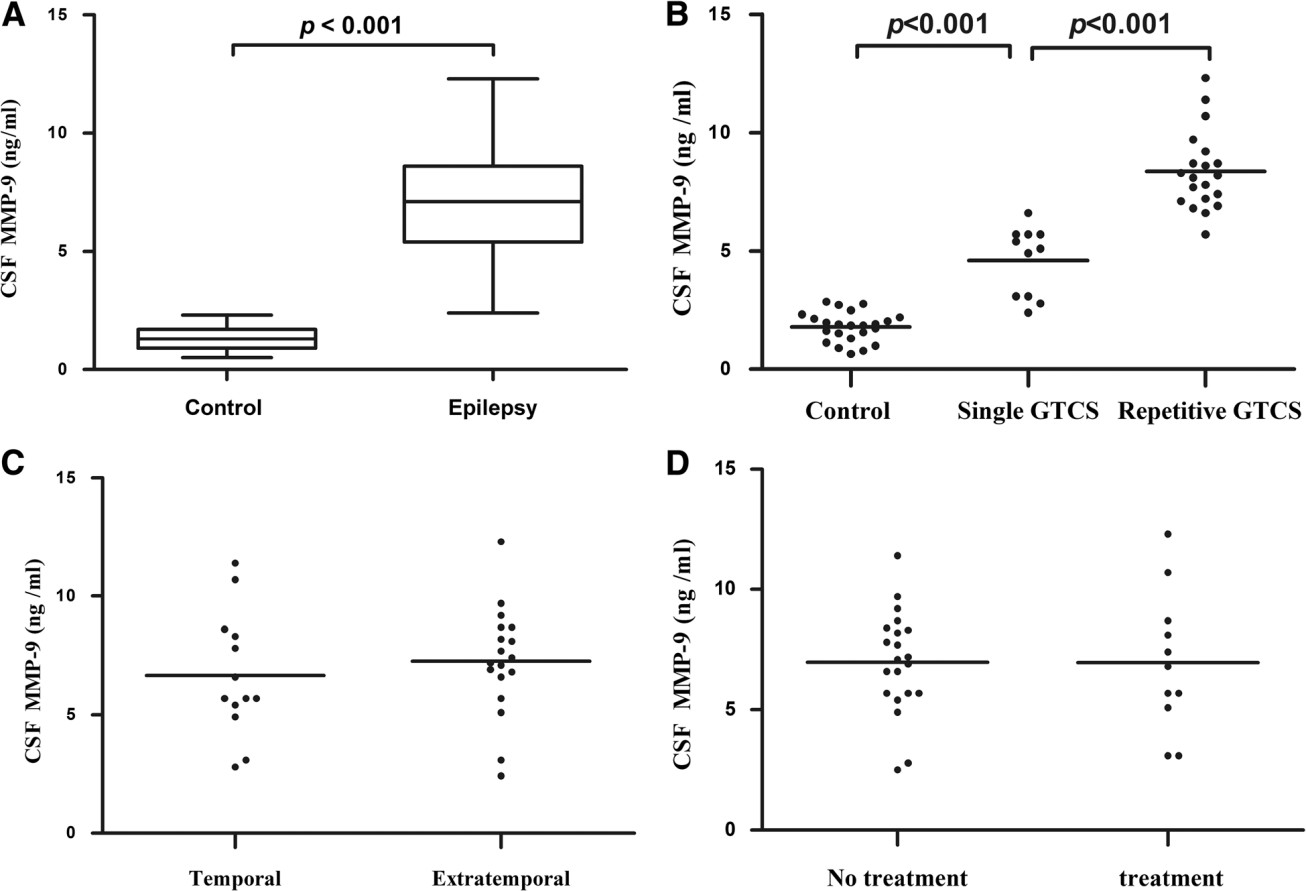
**CSF MMP-9 levels in seizure patients and controls.** (**A**) Boxplot representing the distribution (median and interquartile range) of CSF MMP-9 concentrations for control subjects (Control) and seizure patients (Epilepsy). Significantly higher MMP-9 levels in seizure patients compared with controls are shown. (**B**) Patients with repetitive generalized tonic-clonic seizures tended to have higher CSF MMP-9 levels compared with those who had single generalized tonic-clonic seizure and controls. (**C**) No significant difference found between temporal and extratemporal epilepsy (*P* > 0.05). (**D**) No significant difference between AEDs treatment groups (*P* > 0.05). Horizontal line indicates median. Significant change at *P* < 0.001 using Mann–Whitney U tests.

**Figure 2:**
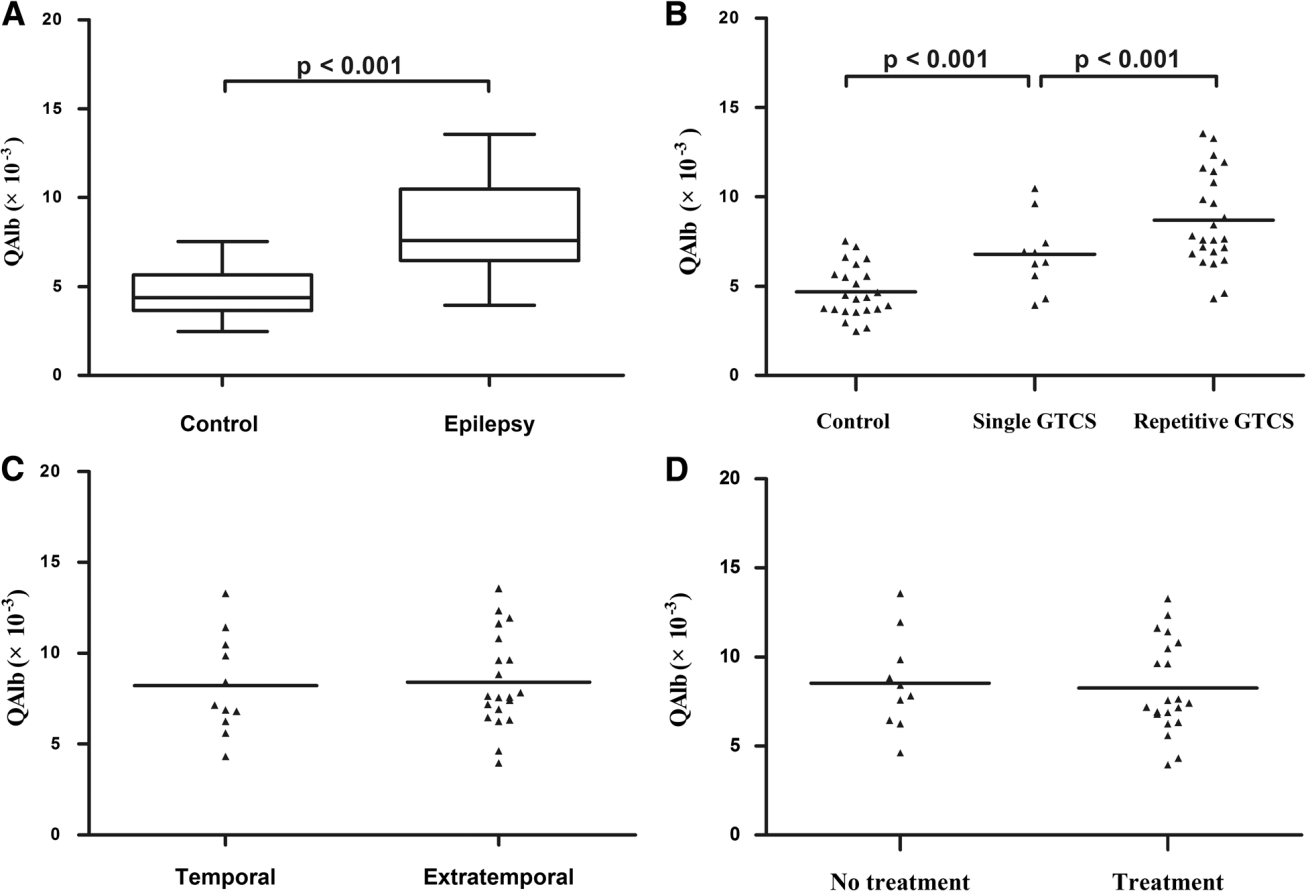
**Mean QAlb values in seizure patients and controls.** (**A**) Boxplot representing the distribution (median and interquartile range) of QAlb for control and epilepsy patients. QAlb values were higher in the epilepsy group than in controls. (**B**) Patients with repetitive GTC seizures had higher QAlb values than those with a single GTC seizure. (**C**) No significant difference found between temporal and extratemporal epilepsy (*P* > 0.05). (**D**) No difference measured between AEDs treatment groups (*P* > 0.05). Horizontal line indicates median; significant *P*-values among groups are displayed (Mann–Whitney test).

We measured significantly higher MMP-9 concentrations (Table 2 and Figure 1A; *P* < 0.001) and QAlb values (Table 2 and Figure 2A) in seizure patients compared with controls. We found significantly higher concentrations of MMP-9 (Figure 1B) and QAlb values (Figure 2B) in RS patients compared with SS and controls (Figure 1B). Analysis of temporal epilepsy and extra-temporal epilepsy showed no significant difference in CSF MMP-9 (Figure 1C) or QAlb values (Figure 2C). In addition, when considering use of anti-epileptic drugs (AEDs), we found no difference in MMP-9 levels (Figure 1D) or QAlb values (Figure 2D). All seizure patients had normal serum protein and albumin concentrations. These data suggest that the CSF albumin increase was regulated by the increased permeability of the damaged BBB.

**Table 2:** The mean concentrations of CSF MMP-9, CSF cell count and albumin, and serum albumin from patients with GTC seizures and controls

**Variable**	**Patient (mean ± SD)**	**Control (mean ± SD)**	***P***
CSF leukocytes (10^6^/l)	8.2 ± 2.3	2.0036 ± 1.4	<0.05
CSF erythrocytes (10^6^/l)	19.6 ± 30.5	14.4 ± 21.8	>0.05
CSF albumin (g/l)	278.6 ± 35.5 (×10^-3^)	198.2 ± 48.7 (×10^-3^)	<0.05
Serum albumin (g/l)	41.6 ± 1.3	42.3 ± 1.5	>0.05
QAlb	8.35 ± 2.6 (×10^-3^)	4.7 ± 1.4 (×10^-3^)	<0.001
MMP-9 (ng /ml)	7.0 ± 2.4	1.80 ± 0.63	<0.001

Patient compared to control by Mann–Whitney test.

### MMP-9 concentrations and QAlb values are correlated

**Figure 3:**
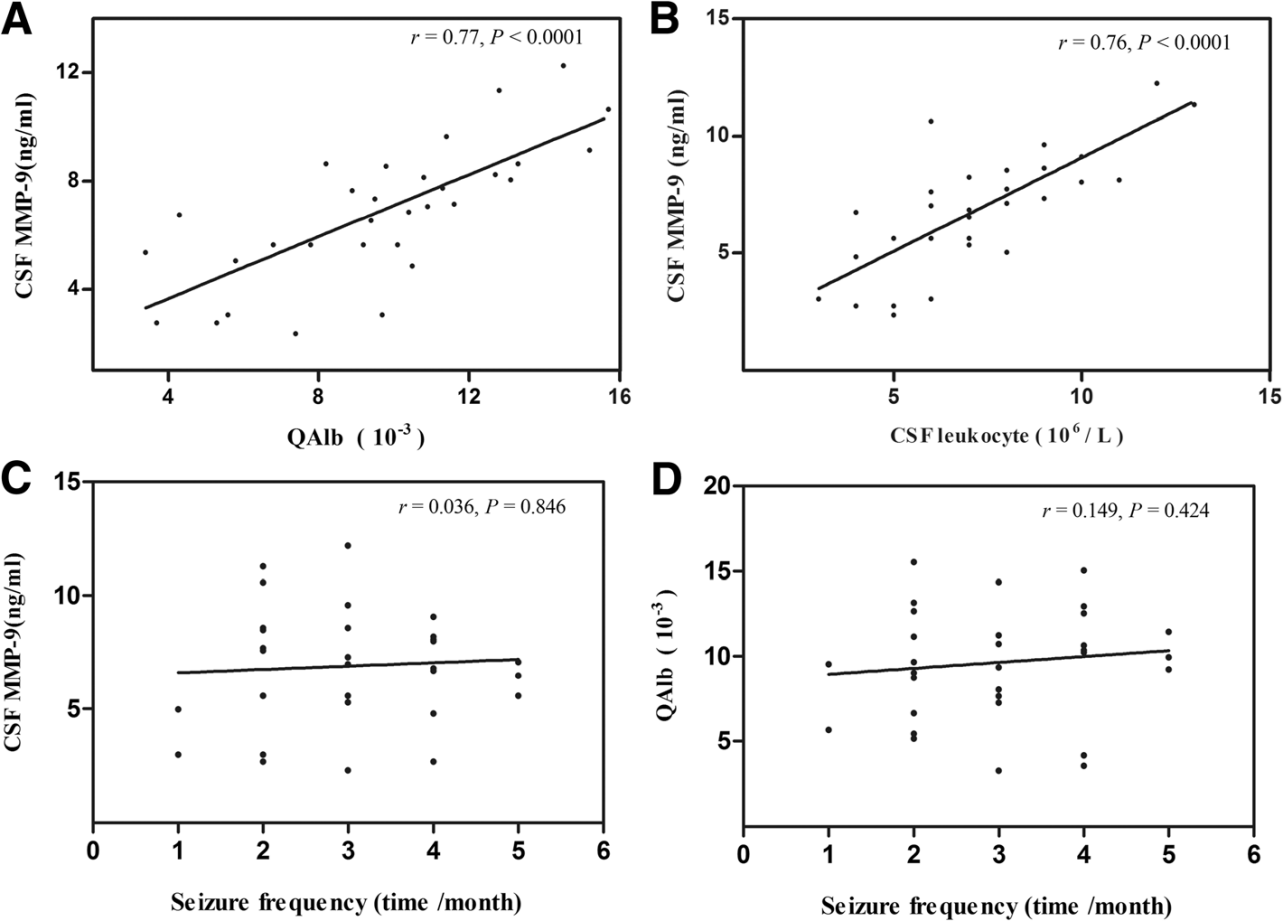
**Correlation analysis of CSF MMP-9 levels in seizure patients.** (**A** )Significant correlation between CSF MMP-9 levels and QAlb value (*P* < 0.0001, *r* = 0.77). (**B**) Significant correlation between CSF MMP-9 levels and CSF leukocyte count (*P* < 0.0001, *r* =0.76). (**C**) No significant correlation between CSF MMP-9 levels and seizure frequency (*P =* 0.846, *r* = 0.036). (**D**) No significant correlation between QAlb value and seizure frequency ( *P =* 0.424, *r* = 0.149). Correlation analysis performed with Spearman’s rank correlation coefficient.

To determine if the increased MMP-9 levels and higher QAlb values were related, we performed correlation analysis. We found a strong correlation between MMP-9 concentration and QAlb values in patients after GTC seizures (Figure 3A, *r* = 0.76, *P* < 0.001). We also found a correlation between CSF MMP-9 concentration and leukocyte counts in patients after GTC seizures (Figure 3B, *r* = 0.77, *P* < 0.001). In fact, our data show the greater number of leukocytes in the CSF aligned with higher MMP-9 concentrations. These results suggest leukocytes are the source of increased MMP-9 levels in CSF after seizure.

We also looked at the correlation between CSF MMP-9 levels and seizure frequency and correlation between QAlb values and seizure frequency. We found that neither CSF MMP-9 levels nor QAlb values correlated with seizure frequency (Figure 3C-D).

### MMP-9 enzyme activity detected in seizure samples

**Figure 4:**
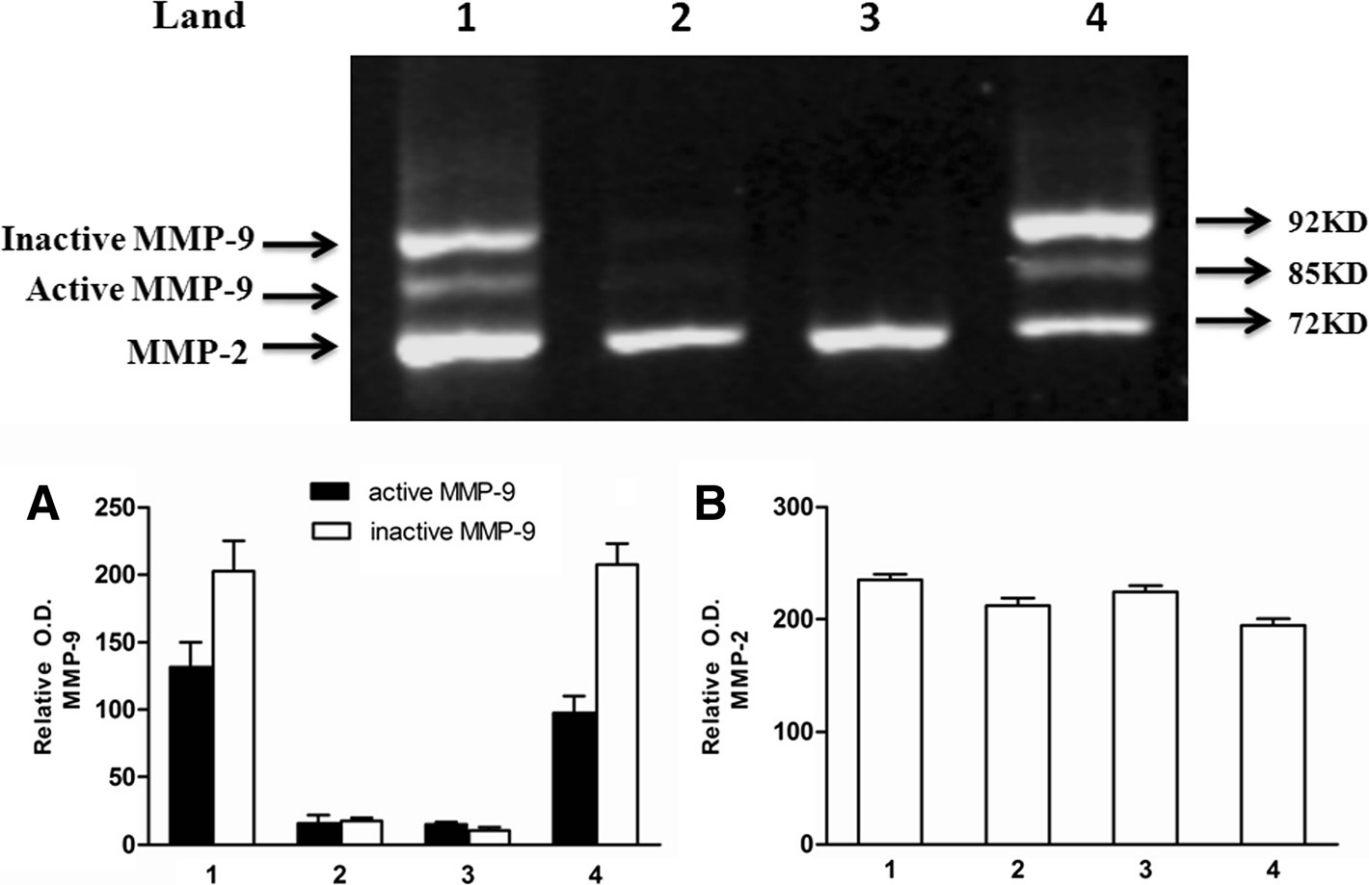
**MMP enzyme activity of CSF samples.** Top panel: Gelatin zymography gel results. Lane 1: GTC seizure sample; lanes 2 and 3 are controls; lane 4: single GTC seizure sample. Note that only seizure samples had positive MMP activity. (**A**) The relative optical density of MMP-9. (**B**) The relative optical density of MMP-2.

We randomly selected CSF specimens from two patients and two controls for enzyme activity analysis by gelatin zymography. As shown in Figure 4, all CSF specimens from patients and controls show a band at molecular mass 72 kD, representing MMP-2. However, only CSF specimens from patients after seizure showed MMP-9 activity. We found that RS and SS samples had a higher ratio of active/inactive MMP-9 activity, supporting the argument that higher MMP-9 levels and the severity of BBB damage are dependent on the number of seizure insults but not MMP-2. Interestingly, polymorphonuclear neutrophils were present in 42% of patient CSF samples. This confirms a transient influx of neutrophils into the CSF of patients after epileptic seizure.

## Discussion

Many questions are unanswered regarding how BBB integrity changes during epileptic seizures. And it is unclear whether elevated CSF MMP-9 contributes to alterations in BBB permeability. Our study addresses these questions by demonstrating, in vivo, a marked dysfunction of the BBB with significantly higher MMP-9 in CSF after epileptic seizures. To our knowledge, this is the first prospective study of the link between CSF MMP-9 and BBB dysfunction in patients after epileptic seizure.

A dysfunctional BBB leads to increased permeability, allowing increased CSF marker passage. Therefore, CSF protein levels are a reliable way to monitor BBB integrity without using invasive methods. A common and well-established assessment of BBB function measures the level of albumin, a blood-specific protein in CSF and serum, reported as the CSF/serum albumin ratio (QAlb) [19,20]. An increase in QAlb reflects the serum albumin leakage in CSF due to BBB dysfunction, especially blood-CSF barrier dysfunction [21-23]. Previous studies have shown increased QAlb is correlated with status epilepticus [7]. In our study, we found the QAlb value was significantly increased in patients after seizure, suggesting BBB dysfunction after epileptic seizure. This finding is consistent with histological studies showing albumin accumulation in human epileptic brains [24,25]. Ultrastructural studies on human resected epileptic tissue also show clear BBB abnormalities, including increased micropinocytosis and the presence of abnormal tight junctions [26,27].

Accumulating experimental evidence indicates BBB dysfunction and inflammatory mediators decrease the threshold for individual seizures and contribute to epileptogenesis. Thus, BBB breakdown is regarded as an important pathophysiological event in seizures and epileptogenesis. Therefore, understanding the mechanisms controlling BBB disruption is critical to the understanding of epileptogenesis and may lead to new therapeutic targets for the prevention and treatment of epilepsy.

Matrix metallopeptidases (MMPs) represent a family of extracellular soluble or membrane bound neutral proteases having complex functions in normal and pathological conditions. Among MMPs, MMP-2 and MMP-9 are the only two gelatinases in humans. MMP-2 is normally found in brain and CSF [28]. In our study we found MMP-2 in the control CSF and in the patient CSF, indicating that expression of CSF MMP-2 in patients was not a result of seizure. MMP-9 is most abundantly expressed in the developing brain and is produced mainly by neurons and, to some extent, by glial cells in the CNS [29-31]. Under normal physiological conditions, MMP-9 is involved in dendritic spine remodeling, synaptic plasticity, learning and memory formation [32-35]. Increasing evidence in experimental animal models of epilepsy and human epileptic brains shows that MMP-9 plays a role in the pathogenesis of epilepsy by contributing to neuronal death, aberrant synaptic plasticity and neuroinflammation [36-39]. One study found that MMP-9 participates in the occurrence of seizures by converting pro-BDNF to mature BDNF in the hippocampus [38]. BDNF contributes to epilepsy in many ways. The roles of BDNF are not only important in acute seizures and epileptogenesis, but are also likely to be important in chronic epilepsy. Increased levels of BDNF exert functional effects that are consistent with a pro-convulsant action. Animal model studies suggest intrahippocampal infusion of BDNF, and transgenic overexpression of BDNF significantly increases seizure susceptibility and severity [40-43]. Other studies have reported MMP-9-induced cell death is closely linked with the pathogenesis of epilepsy after SE [34,44]. In particular, MMP-9 knockout mice were less susceptible to seizure-induced brain injury, and MMP-9 inhibition ameliorated cell death following pilocarpine-induced seizures in infant rats [34]. Further, MMP-9 plays an important role in epileptogeness mainly due to MMP-9-related synaptic plasticity changes rather than neuronal death [45]. However, the relationship between CSF MMP-9 and BBB dysfunction in patients after epileptic seizures remains unclear.

A growing body of experimental and clinical evidence shows MMP-9 plays a major role in BBB disruption in a variety of pathological conditions including CNS infections [46-48], stroke [12,15,49,50], multiple sclerosis [51] and traumatic brain injury [52]. MMP-9 cleaves type IV collagen, a major component of the basement membrane of the cerebral epithelium that is responsible for the integrity of the BBB. MMP-9 also degrades a number of other extracellular matrix molecules including type V and XI collagens, laminin and aggrecan core protein [29,53,54]. Our study confirms a strong correlation between CSF MMP-9 levels and BBB dysfunction in patients after GTC seizures and argues MMP-9, not MMP-2, plays a role in BBB disruption during seizures.

Here, we also confirmed a transient influx of granulocytes into CSF of patients after epileptic seizure. Neutrophils in CSF samples are commonly considered a pathological feature. The migration of leukocytes from the bloodstream into the CNS is a key event in the pathogenesis of inflammatory neurological diseases. Recent data suggest that leukocyte-endothelial interaction and brain infiltration by leukocytes plays an important role in the pathogenesis of epilepsy [55-57]. In our study, we also found that elevated MMP-9 levels in CSF are primarily related to the number of immigrated leukocytes, with positive correlation between CSF MMP-9 levels and CSF leukocytes as well as between CSF MMP-9 levels and QAlb values. Leukocytes contribute to seizure pathogenesis acutely through affects on the BBB and chronically through several mechanisms including generation of oxygen free radicals, release of cytotoxic enzymes, vascular alterations and increase in cytokine and chemoattractant release [58]. Thus, we confirm that increased CSF MMP-9 represents the accumulation of activated leukocytes. It is reasonable to suspect that leukocytes are major producers of MMP-9. Furthermore, it has been demonstrated that injured neuronal and glial cells are major sources of MMP-9, supporting the neuroinflammatory response by releasing cytokines during epileptic seizures [36,37,53]. Indeed, we observed MMP-9 activity in CSF specimens from patients with seizures only.

We also analyzed effects of antiepileptic drugs (AEDs) on CSF MMP-9 concentrations and found no significant MMP-9 changes in patients with seizure. It is important to note several other reports show AEDs inhibit MMP-9 production and protect BBB function in epileptic patients and cerebral ischemic rats [59,60]. It has been proposed that MMP-9 is involved in aberrant synaptic formation in hippocampi of patients with temporal lobe epilepsy [39]. Therefore, we also analyzed temporal and extra-temporal seizures. Our findings show no differences in CSF MMP-9 levels between patients with temporal seizures and patients with extra-temporal seizures. In general, our findings argue MMP-9 overexpression is associated with activation of leukocytes, most likely polymorphonuclear, that serve as a key cellular source of MMP-9 in CSF. In turn, this promotes leukocyte recruitment, causing BBB breakdown, microvascular basal lamina proteolysis, and ultimately contributes to neuronal injury after epileptic seizure.

## Conclusions

In this study, we found a significant correlation between CSF MMP-9 concentrations and CSF leukocyte counts, leading to BBB dysfunction. We suggest increased concentrations of MMP-9 in CSF were partly derived from leukocytes. These results demonstrate that seizure is characterized by invasion of leukocytes into the CSF and increased CSF MMP-9 levels are associated with BBB dysfunction in patients with seizures.

## Competing interests

The authors declared that they have no competing interest.

## Authors’ contributions

YJL designed the research, ZHW reviewed and helped in analyzing data, BZ, XZ, MJW and STS processed serum and CSF from the patients and controls, JB, TL, CJG, SJZ, XLK, XZ and HZ performed the research, and YJL and ZHW wrote the paper. All authors read and approved the final manuscript.

## References

[B1] Abbott NJ Ronnback L Hansson E Astrocyte-endothelial interactions at the blood–brain barrier Nat Rev Neurosci 2006 7 41 53 10.1038/nrn1824 16371949

[B2] Hawkins BT Davis TP The blood–brain barrier/neurovascular unit in health and disease Pharmacol Rev 2005 57 173 185 10.1124/pr.57.2.4 15914466

[B3] Tomkins O Shelef I Kaizerman I Eliushin A Afawi Z Misk A Gidon M Cohen A Zumsteg D Friedman A Blood–brain barrier disruption in post-traumatic epilepsy J Neurol Neurosurg Psychiatry 2008 79 774 777 10.1136/jnnp.2007.126425 17991703

[B4] van Vliet EA da Costa AS Redeker S van Schaik R Aronica E Gorter JA Blood–brain barrier leakage may lead to progression of temporal lobe epilepsy Brain 2007 130 521 534 10.1093/brain/awl318 17124188

[B5] Korn A Golan H Melamed I Pascual-Marqui R Friedman A Focal cortical dysfunction and blood–brain barrier disruption in patients with postconcussion syndrome J Clin Neurophysiol 2005 22 1 9 10.1097/01.WNP.0000150973.24324.A7 15689708

[B6] Strbian D Durukan A Pitkonen M Marinkovic I Tatlisumak E Pedrono E Abo-Ramadan U Tatlisumak T The blood–brain barrier is continuously open for several weeks following transient focal cerebral ischemia Neuroscience 2008 153 175 181 10.1016/j.neuroscience.2008.02.012 18367342

[B7] Correale J Rabinowicz AL Heck CN Smith TD Loskota WJ DeGiorgio CM Status epilepticus increases CSF levels of neuron-specific enolase and alters the blood–brain barrier Neurology 1998 50 1388 1391 10.1212/WNL.50.5.1388 9595992

[B8] Friedman A Blood–brain barrier dysfunction, status epilepticus, seizures, and epilepsy: a puzzle of a chicken and egg? Epilepsia 2011 52 Suppl 8 19 20 21967353 10.1111/j.1528-1167.2011.03227.x PMC3234990

[B9] Sahin D Ilbay G Ates N Changes in the blood–brain barrier permeability and in the brain tissue trace element concentrations after single and repeated pentylenetetrazole-induced seizures in rats Pharmacol Res 2003 48 69 73 12770517

[B10] Marchi N Angelov L Masaryk T Fazio V Granata T Hernandez N Hallene K Diglaw T Franic L Najm I Janigro D Seizure-promoting effect of blood–brain barrier disruption Epilepsia 2007 48 732 742 10.1111/j.1528-1167.2007.00988.x 17319915 PMC4135474

[B11] Marchi N Tierney W Alexopoulos AV Puvenna V Granata T Janigro D The etiological role of blood–brain barrier dysfunction in seizure disorders Cardiovasc Psychiatry Neurol 2011 2011 482415 21541221 10.1155/2011/482415 PMC3085334

[B12] Barr TL Latour LL Lee KY Schaewe TJ Luby M Chang GS El-Zammar Z Alam S Hallenbeck JM Kidwell CS Warach S Blood–brain barrier disruption in humans is independently associated with increased matrix metalloproteinase-9 Stroke 2010 41 e123 e128 10.1161/STROKEAHA.109.570515 20035078 PMC2827673

[B13] Aoki T Sumii T Mori T Wang X Lo EH Blood–brain barrier disruption and matrix metalloproteinase-9 expression during reperfusion injury: mechanical versus embolic focal ischemia in spontaneously hypertensive rats Stroke 2002 33 2711 2717 10.1161/01.STR.0000033932.34467.97 12411666

[B14] Jin R Yang G Li G Molecular insights and therapeutic targets for blood–brain barrier disruption in ischemic stroke: critical role of matrix metalloproteinases and tissue-type plasminogen activator Neurobiol Dis 2010 38 376 385 10.1016/j.nbd.2010.03.008 20302940 PMC2862862

[B15] Gidday JM Gasche YG Copin JC Shah AR Perez RS Shapiro SD Chan PH Park TS Leukocyte-derived matrix metalloproteinase-9 mediates blood–brain barrier breakdown and is proinflammatory after transient focal cerebral ischemia Am J Physiol Heart Circ Physiol 2005 289 H558 H568 10.1152/ajpheart.01275.2004 15764676

[B16] Tsai HC Chung LY Chen ER Liu YC Lee SS Chen YS Sy CL Wann SR Yen CM Association of matrix metalloproteinase-9 and tissue inhibitors of metalloproteinase-4 in cerebrospinal fluid with blood–brain barrier dysfunction in patients with eosinophilic meningitis caused by Angiostrongylus cantonensis Am J Trop Med Hyg 2008 78 20 27 18187780

[B17] Fernandez-Lopez D Faustino J Daneman R Zhou L Lee SY Derugin N Wendland MF Vexler ZS Blood–brain barrier permeability is increased after acute adult stroke but not neonatal stroke in the rat J Neurosci 2012 32 9588 9600 10.1523/JNEUROSCI.5977-11.2012 22787045 PMC3539825

[B18] Berg AT Scheffer IE New concepts in classification of the epilepsies: entering the 21st century Epilepsia 2011 52 1058 1062 10.1111/j.1528-1167.2011.03101.x 21635233

[B19] Tibbling G Link H Ohman S Principles of albumin and IgG analyses in neurological disorders. I. Establishment of reference values Scand J Clin Lab Invest 1977 37 385 390 10.3109/00365517709091496 337459

[B20] Ganrot K Laurell CB Measurement of IgG and albumin content of cerebrospinal fluid, and its interpretation Clin Chem 1974 20 571 573 4207912

[B21] Chalbot S Zetterberg H Blennow K Fladby T Andreasen N Grundke-Iqbal I Iqbal K Blood-cerebrospinal fluid barrier permeability in Alzheimer's disease J Alzheimers Dis 2011 25 505 515 21471645 10.3233/JAD-2011-101959 PMC3139450

[B22] Sindic CJ Van Antwerpen MP Goffette S The intrathecal humoral immune response: laboratory analysis and clinical relevance Clin Chem Lab Med 2001 39 333 340 11388658 10.1515/CCLM.2001.052

[B23] Reiber H Padilla-Docal B Jensenius JC Dorta-Contreras AJ Mannan-binding lectin in cerebrospinal fluid: a leptomeningeal protein Fluids Barriers CNS 2012 9 17 10.1186/2045-8118-9-17 22889364 PMC3487976

[B24] Raabe A Schmitz AK Pernhorst K Grote A von der Brelie C Urbach H Friedman A Becker AJ Elger CE Niehusmann P Cliniconeuropathologic correlations show astroglial albumin storage as a common factor in epileptogenic vascular lesions Epilepsia 2012 53 539 548 10.1111/j.1528-1167.2012.03405.x 22372630 PMC3669690

[B25] Marchi N Teng Q Ghosh C Fan Q Nguyen MT Desai NK Bawa H Rasmussen P Masaryk TK Janigro D Blood–brain barrier damage, but not parenchymal white blood cells, is a hallmark of seizure activity Brain Res 2010 1353 176 186 20599815 10.1016/j.brainres.2010.06.051 PMC2933328

[B26] Cornford EM Epilepsy and the blood–brain barrier: endothelial cell responses to seizures Adv Neurol 1999 79 845 862 10514868

[B27] Heinemann U Kaufer D Friedman A Blood–brain barrier dysfunction, TGFbeta signaling, and astrocyte dysfunction in epilepsy Glia 2012 60 1251 1257 10.1002/glia.22311 22378298 PMC3615248

[B28] Grossetete M Phelps J Arko L Yonas H Rosenberg GA Elevation of matrix metalloproteinases 3 and 9 in cerebrospinal fluid and blood in patients with severe traumatic brain injury Neurosurgery 2009 65 702 708 10.1227/01.NEU.0000351768.11363.48 19834375 PMC2764327

[B29] Dong X Song YN Liu WG Guo XL Mmp-9, a potential target for cerebral ischemic treatment Curr Neuropharmacol 2009 7 269 275 10.2174/157015909790031157 20514206 PMC2811860

[B30] Michaluk P Kaczmarek L Matrix metalloproteinase-9 in glutamate-dependent adult brain function and dysfunction Cell Death Differ 2007 14 1255 1258 10.1038/sj.cdd.4402141 17431423

[B31] Szklarczyk A Lapinska J Rylski M McKay RD Kaczmarek L Matrix metalloproteinase-9 undergoes expression and activation during dendritic remodeling in adult hippocampus J Neurosci 2002 22 920 930 11826121 10.1523/JNEUROSCI.22-03-00920.2002 PMC6758472

[B32] Michaluk P Wawrzyniak M Alot P Szczot M Wyrembek P Mercik K Medvedev N Wilczek E De Roo M Zuschratter W Influence of matrix metalloproteinase MMP-9 on dendritic spine morphology J Cell Sci 2011 124 3369 3380 10.1242/jcs.090852 21896646

[B33] Wang XB Bozdagi O Nikitczuk JS Zhai ZW Zhou Q Huntley GW Extracellular proteolysis by matrix metalloproteinase-9 drives dendritic spine enlargement and long-term potentiation coordinately Proc Natl Acad Sci U S A 2008 105 19520 19525 10.1073/pnas.0807248105 19047646 PMC2614793

[B34] Hoehna Y Uckermann O Luksch H Stefovska V Marzahn J Theil M Gorkiewicz T Gawlak M Wilczynski GM Kaczmarek L Ikonomidou C Matrix metalloproteinase 9 regulates cell death following pilocarpine-induced seizures in the developing brain Neurobiol Dis 2012 48 339 347 10.1016/j.nbd.2012.06.023 22782080

[B35] Nagy V Bozdagi O Matynia A Balcerzyk M Okulski P Dzwonek J Costa RM Silva AJ Kaczmarek L Huntley GW Matrix metalloproteinase-9 is required for hippocampal late-phase long-term potentiation and memory J Neurosci 2006 26 1923 1934 10.1523/JNEUROSCI.4359-05.2006 16481424 PMC4428329

[B36] Konopka A Grajkowska W Ziemianska K Roszkowski M Daszkiewicz P Rysz A Marchel A Koperski L Wilczynski GM Dzwonek J Matrix metalloproteinase-9 (MMP-9) in human intractable epilepsy caused by focal cortical dysplasia Epilepsy Res 2013 104 45 58 10.1016/j.eplepsyres.2012.09.018 23182966

[B37] Li S Yu S Zhang C Shu H Liu S An N Yang M Yin Q Yang H Increased expression of matrix metalloproteinase 9 in cortical lesions from patients with focal cortical dysplasia type IIb and tuberous sclerosis complex Brain Res 2012 1453 46 55 22459050 10.1016/j.brainres.2012.03.009

[B38] Mizoguchi H Nakade J Tachibana M Ibi D Someya E Koike H Kamei H Nabeshima T Itohara S Takuma K Matrix metalloproteinase-9 contributes to kindled seizure development in pentylenetetrazole-treated mice by converting pro-BDNF to mature BDNF in the hippocampus J Neurosci 2011 31 12963 12971 10.1523/JNEUROSCI.3118-11.2011 21900575 PMC6623408

[B39] Wilczynski GM Konopacki FA Wilczek E Lasiecka Z Gorlewicz A Michaluk P Wawrzyniak M Malinowska M Okulski P Kolodziej LR Important role of matrix metalloproteinase 9 in epileptogenesis J Cell Biol 2008 180 1021 1035 10.1083/jcb.200708213 18332222 PMC2265409

[B40] Lahteinen S Pitkanen A Koponen E Saarelainen T Castren E Exacerbated status epilepticus and acute cell loss, but no changes in epileptogenesis, in mice with increased brain-derived neurotrophic factor signaling Neuroscience 2003 122 1081 1092 10.1016/j.neuroscience.2003.08.037 14643774

[B41] Scharfman HE Goodman JH Sollas AL Croll SD Spontaneous limbic seizures after intrahippocampal infusion of brain-derived neurotrophic factor Exp Neurol 2002 174 201 214 10.1006/exnr.2002.7869 11922662

[B42] Croll SD Suri C Compton DL Simmons MV Yancopoulos GD Lindsay RM Wiegand SJ Rudge JS Scharfman HE Brain-derived neurotrophic factor transgenic mice exhibit passive avoidance deficits, increased seizure severity and in vitro hyperexcitability in the hippocampus and entorhinal cortex Neuroscience 1999 93 1491 1506 10.1016/S0306-4522(99)00296-1 10501474 PMC2504500

[B43] Xu B Michalski B Racine RJ Fahnestock M The effects of brain-derived neurotrophic factor (BDNF) administration on kindling induction, Trk expression and seizure-related morphological changes Neuroscience 2004 126 521 531 10.1016/j.neuroscience.2004.03.044 15183502

[B44] Kim GW Kim HJ Cho KJ Kim HW Cho YJ Lee BI The role of MMP-9 in integrin-mediated hippocampal cell death after pilocarpine-induced status epilepticus Neurobiol Dis 2009 36 169 180 10.1016/j.nbd.2009.07.008 19631748

[B45] Takacs E Nyilas R Szepesi Z Baracskay P Karlsen B Rosvold T Bjorkum AA Czurko A Kovacs Z Kekesi AK Juhasz G Matrix metalloproteinase-9 activity increased by two different types of epileptic seizures that do not induce neuronal death: a possible role in homeostatic synaptic plasticity Neurochem Int 2010 56 799 809 10.1016/j.neuint.2010.03.003 20303372

[B46] Paul R Lorenzl S Koedel U Sporer B Vogel U Frosch M Pfister HW Matrix metalloproteinases contribute to the blood–brain barrier disruption during bacterial meningitis Ann Neurol 1998 44 592 600 10.1002/ana.410440404 9778257

[B47] Matsuura E Umehara F Hashiguchi T Fujimoto N Okada Y Osame M Marked increase of matrix metalloproteinase 9 in cerebrospinal fluid of patients with fungal or tuberculous meningoencephalitis J Neurol Sci 2000 173 45 52 10.1016/S0022-510X(99)00303-2 10675579

[B48] Brown HC Chau TT Mai NT Day NP Sinh DX White NJ Hien TT Farrar J Turner GD Blood–brain barrier function in cerebral malaria and CNS infections in Vietnam Neurology 2000 55 104 111 10.1212/WNL.55.1.104 10891914

[B49] Guo M Cox B Mahale S Davis W Carranza A Hayes K Sprague S Jimenez D Ding Y Pre-ischemic exercise reduces matrix metalloproteinase-9 expression and ameliorates blood–brain barrier dysfunction in stroke Neuroscience 2008 151 340 351 10.1016/j.neuroscience.2007.10.006 18160227

[B50] Wang Z Meng CJ Shen XM Shu Z Ma C Zhu GQ Liu HX He WC Sun XB Huo L Potential contribution of hypoxia-inducible factor-1alpha, aquaporin-4, and matrix metalloproteinase-9 to blood–brain barrier disruption and brain edema after experimental subarachnoid hemorrhage J Mol Neurosci 2012 48 273 280 10.1007/s12031-012-9769-6 22528459

[B51] Fainardi E Castellazzi M Bellini T Manfrinato MC Baldi E Casetta I Paolino E Granieri E Dallocchio F Cerebrospinal fluid and serum levels and intrathecal production of active matrix metalloproteinase-9 (MMP-9) as markers of disease activity in patients with multiple sclerosis Mult Scler 2006 12 294 301 10.1191/135248506ms1274oa 16764342

[B52] Higashida T Kreipke CW Rafols JA Peng C Schafer S Schafer P Ding JY Dornbos D 3rd Li X Guthikonda M The role of hypoxia-inducible factor-1alpha, aquaporin-4, and matrix metalloproteinase-9 in blood–brain barrier disruption and brain edema after traumatic brain injury J Neurosurg 2011 114 92 101 10.3171/2010.6.JNS10207 20617879

[B53] Yin P Yang L Zhou HY Sun RP Matrix metalloproteinase-9 may be a potential therapeutic target in epilepsy Med Hypotheses 2011 76 184 186 10.1016/j.mehy.2010.09.013 20888701

[B54] Ralay Ranaivo H Hodge JN Choi N Wainwright MS Albumin induces upregulation of matrix metalloproteinase-9 in astrocytes via MAPK and reactive oxygen species-dependent pathways J Neuroinflammation 2012 9 68 10.1186/1742-2094-9-68 22507553 PMC3419618

[B55] Silverberg J Ginsburg D Orman R Amassian V Durkin HG Stewart M Lymphocyte infiltration of neocortex and hippocampus after a single brief seizure in mice Brain Behav Immun 2010 24 263 272 10.1016/j.bbi.2009.10.006 19822204

[B56] Zattoni M Mura ML Deprez F Schwendener RA Engelhardt B Frei K Fritschy JM Brain infiltration of leukocytes contributes to the pathophysiology of temporal lobe epilepsy J Neurosci 2011 31 4037 4050 10.1523/JNEUROSCI.6210-10.2011 21411646 PMC6623535

[B57] Fabene PF Navarro Mora G Martinello M Rossi B Merigo F Ottoboni L Bach S Angiari S Benati D Chakir A A role for leukocyte-endothelial adhesion mechanisms in epilepsy Nat Med 2008 14 1377 1383 10.1038/nm.1878 19029985 PMC2710311

[B58] Fabene PF Laudanna C Constantin G Leukocyte trafficking mechanisms in epilepsy Mol Immunol 2013 55 100 104 10.1016/j.molimm.2012.12.009 23351392

[B59] Takahashi Y Imai K Ikeda H Kubota Y Yamazaki E Susa F Open study of pranlukast add-on therapy in intractable partial epilepsy Brain Dev 2013 35 236 244 10.1016/j.braindev.2012.04.001 22571867

[B60] Wang Z Leng Y Tsai LK Leeds P Chuang DM Valproic acid attenuates blood–brain barrier disruption in a rat model of transient focal cerebral ischemia: the roles of HDAC and MMP-9 inhibition J Cereb Blood Flow Metab 2011 31 52 57 10.1038/jcbfm.2010.195 20978517 PMC3049473

